# Placing equity at the heart of eHealth implementation: a qualitative pilot study

**DOI:** 10.1186/s12939-022-01640-5

**Published:** 2022-03-18

**Authors:** Milena Heinsch, Campbell Tickner, Frances Kay-Lambkin

**Affiliations:** Centre for Brain and Mental Health Research, University of Newcastle, University Drive, Callaghan, NSW Australia

**Keywords:** Implementation, eHealth, Digital health, Equity, Social justice, Theory

## Abstract

**Background:**

There is a growing urgency to tackle issues of equity and justice in the implementation of eHealth technologies.

**Methods:**

Qualitative interviews were conducted with 19 multidisciplinary health professionals to explore the implementation and uptake of eHealth technologies in practice. The aim of this article was to examine in more detail issues of equity and justice in the implementation and uptake of eHealth technologies in practice. Results were analysed using Braun and Clarke’s six-step reflexive thematic analysis approach.

**Results:**

Nancy Fraser’s concept of social justice is introduced as a novel framework for inquiry into the implementation of digital health services. Health professionals reported that eHealth offered their clients a greater sense of safety, convenience, and flexibility, allowing them to determine the nature and pace of their healthcare, and giving them more control over their treatment and recovery. However, they also expressed concerns about the use of eHealth with clients whose home environment is unsafe. Application of Fraser’s framework revealed that eHealth technologies may not always provide a secure clinical space in which the voices of vulnerable clients can be *recognised* and heard. It also highlighted critical systemic and cultural barriers that hinder the *representation* of clients’ voices in the decision to use eHealth technologies and perpetuate inequalities in the *distribution* of eHealth services.

**Conclusions:**

To facilitate broad participation, eHealth tools need to be adaptable to the needs and circumstances of diverse groups. Future implementation science efforts must also be directed at identifying and addressing the underlying structures that hinder equitable recognition, representation, and distribution in the implementation of eHealth resources.

**Supplementary Information:**

The online version contains supplementary material available at 10.1186/s12939-022-01640-5.

## Background

In 2021, Brownson and colleagues published an article in *Implementation Science* that proposed a bold vision for making health equity the highest priority in the field. While recognising the longstanding emphasis of implementation science on health disparities (health differences that are closely linked with economic, social, or environmental disadvantage [1]), the authors argue that a more explicit commitment to health equity (the principle underlying a moral commitment to reduce—and, ultimately, eliminate—disparities in health [1]) is increasingly urgent [2]. These sentiments have been mirrored in a number of recent articles which have called for a stronger focus by implementation science on addressing health inequities [3–8] and a more comprehensive unification of implementation science and health disparities research to accelerate progress toward achieving health equity goals [4].

An area in which a focus on health equity is particularly timely is in the implementation of digital health or eHealth (a term with contested definitions [9] but, broadly speaking, “health services and information delivered or enhanced through the internet and related technologies” [9, 10]). According to the World Health Organisation [11], eHealth has the potential to bring about significant improvements in service equity. Indeed, COVID-19 highlighted the critical role of eHealth (including telehealth) in offering immediate support in times of need, achieving positive health outcomes and enhancing safety and access for patients and clinicians throughout the pandemic [12–14]. Consequently, eHealth, and particularly telehealth (defined as the use of telecommunications technologies to deliver health-related services and information [15];) are now being implemented in health services at an unprecedented rate, supported by government initiatives aimed at facilitating access to consultation services throughout and beyond the pandemic [14].

While the surge in interest and enthusiasm about eHealth represents an exciting opportunity to increase access to high quality healthcare, concerns have been raised that widespread implementation of eHealth technologies may unintentionally perpetuate healthcare disparities for vulnerable and under-resourced groups [16]. Evidence suggests that many people who already experience disadvantage and poor health outcomes are also more likely to face digital exclusion [17]. To reduce these disparities and avoid further disadvantage, careful consideration of health equity is needed “at all stages” of eHealth implementation [18]. Yet, while the wider eHealth literature has repeatedly highlighted issues of unequal access, challenges, and outcomes related to the use of digital health technologies [19–21], to date many eHealth implementation studies have not explicitly drawn on principles or frameworks of equity and justice. Indeed, our recent systematic review of theories informing eHealth implementation identified an absence of theoretical frameworks that specifically emphasise questions of equity and justice in the implementation of eHealth technologies [22]. To ensure digital health solutions can achieve tangible benefits for disadvantaged populations that might help to rectify existing injustices, it is crucial to consider issues of equity and justice in the distribution and use of these solutions.

In this paper, we report the results of the eCliPSE (electronic Clinical Pathways to Service Excellence) qualitative pilot study, which explored the perspectives of multidisciplinary health professionals on the implementation and uptake of eHealth (including telehealth) in practice. While this study did not explicitly set out to examine issues of equity and justice, thematic analysis of participant responses revealed that these issues were interwoven with the developed themes. We therefore chose to explore these issues in more detail, reporting on a subset of findings with the aim of illuminating issues of equity and justice in the implementation and uptake of eHealth technologies in practice. Broader findings from this study will be published elsewhere.

In discussing the findings of this study, we introduce Nancy Fraser’s [23, 24] multidimensional conception of social justice to identify key dimensions and patterns of *distribution*, *recognition*, and *representation* in the implementation of digital health services. Fraser has illuminated how social justice depends not only upon the *distribution* of goods and services, but also on the patterns of recognition and representation in society [23, 24]. Someone can be systematically disadvantaged, and substantially so, if their identity is not *recognised* or valued, or if they do not have a meaningful chance to have their voice *represented* in the social structures and cultures within which they pursue their lives. Implications for facilitating equity and justice in the implementation of eHealth technologies are considered. Findings from this study will inform the refinement of the Integrated Translation and Engagement (ITEM) model designed by the authors to drive the implementation of eCliPSE into mental health and alcohol/other drug use services.

## Methods

A qualitative methodology involving semi-structured interviews was considered most suitable for exploring the complex relationships and interdependencies between variables that characterise the implementation of eHealth technologies [25]. The methods are reported using the Consolidated criteria for Reporting Qualitative research (COREQ [26]; see Additional File 1). Ethics approval was granted for this study in September 2020 (2020/ETH01176).

### Sampling and recruitment

Participants were eligible to take part in the study if they were i) aged 18 years or over, and ii) employed as a clinician or practice manager in a hospital, community-based health service, mental health service, or drug and alcohol service. Participants were recruited from a local health district within New South Wales. A decision was made not to identify the specific district to avoid compromising the anonymity of participants. We sought to include of a diverse range of multidisciplinary service providers to ensure variance in perspectives, skills, knowledge, and demographic composition. Participants were recruited in two ways. First, email invitations were sent to service managers and clinicians across the district, along with a request to share the invitation with other clinicians. Second, the study was advertised on the research teams’ social media accounts (Facebook and Twitter). In total, 19 health professionals agreed to take part in the study; a sample size that is consistent with comparable research [27–29].

### Participants

Interviews were conducted with 19 participants. Table 1 provides an overview of their demographic details. Professions represented were social work (*n* = 10, including two service managers), nursing (*n* = 4), dietetics (*n* = 2), psychiatry (*n* = 1), psychology (*n* = 1), and multicultural liaison (*n* = 1). Participants worked in hospital settings (*n* = 10), mental health services (*n* = 8), and drug and alcohol services (*n* = 1). Participants were also asked to rate their self-perceived experience level with eHealth services and applications. They rated themselves as either a “beginner” (*n* = 7), “adequately experienced” (*n* = 7), “experienced” (*n* = 4), and “highly experienced” (*n* = 1).

**Table 1:** Participant demographics

Demographic Questions		Number of Participants
**Gender**	Female	16
	Male	3
**Age**	Age range	24–60
	Mean age (SD)	44.3 (11.26)
**Health service**	Hospital	10
	Community health service	0
	Mental health service	8
	Drug and alcohol service	1
**Current role**	Social worker	10
	Nurse	4
	Dietitian	2
	Psychiatrist	1
	Psychologist	1
	Multicultural liaison	1
**eHealth experience**	Highly experienced	1
	Experienced	4
	Adequately experienced	7
	Beginner	7
	Do not use eHealth	0

### Data collection

Participants took part in a 40–60-min audio-recorded semi-structured interview, either via telephone (*n* = 14), via videoconferencing technology (*n* = 4) or in-person (*n* = 1). Most interviews were conducted remotely as COVID-19 pandemic restrictions prohibited face-to-face interaction. Interviews were conducted by the second author, who is a female qualified social worker and researcher with experience in qualitative methods. Some participants were known professional acquaintances of the interviewer.

Each interview began with the following question: “can you tell me about your current role and practice?” This question was intentionally kept broad to allow for spontaneous discussion about the role of eHealth in practice and elicit background information to inform the subsequent interview. A semi-structured interview schedule (see Additional File 2) was used to guide each interview, which included questions about participants’ current use of eHealth in practice, organisational support for eHealth implementation, and training and resource needs to support implementation and uptake of eHealth technologies. The interview schedule was used flexibly to accommodate participants’ varying levels of eHealth experience and ensure the collection of rich and detailed data.

### Data analysis

Interviews were audio recorded and transcribed verbatim by a professional transcription service. Data were analysed using Braun and Clarke’s [30, 31] reflexive thematic analysis approach. This approach involves six ‘fluid’ phases that ultimately produce a set of themes [32, 33]. Themes were not viewed as ‘real things’ waiting to be discovered by searching the data [33], but as patterns of shared meaning constructed through the lens of the researcher; their theoretical assumptions, analytic resources and skills, and the data itself [31]. We approached the data from a critical realist perspective, which emphasises that ontology (the nature of reality) cannot be reduced to epistemology (our knowledge of reality) [34, 35], but is mediated through multiple social realities and cultures [36]. Critical realism, thus, allowed us to acknowledge that experiences of social equity—and inequity—are real and true, while also recognising that this reality is shaped by diverse cultural contexts and social systems.

We began by familiarising ourselves with the data, reading and re-reading the transcripts and making notes of initial observations. The second author led the process of coding, starting with three transcripts and coding them inductively to discuss initial codes and ideas with the co-authors. All transcripts were then coded in full using NVivo software to manage the data. During this process, we found that issues of justice and equity were interwoven within several developed codes. We therefore made the decision to examine these issues more closely as we developed themes from the data. Theme development was led by the second author in consultation with the first author. This process led to the production of four key themes: i) a sense of safety and control, ii) expanding the breadth of care, iii) resources and capacity, and iv) a fundamental shift. These themes are presented below with accompanying illustrative quotes from the data.

## Results

When asked to describe their role, only four participants spontaneously discussed eHealth as part of their practice, and three spoke exclusively about the use of telehealth. Even when prompted to consider other eHealth technologies, such as telephone applications and online programs, participants tended to focus on telehealth as the main form of eHealth used in their practice. Further, the use of telehealth was primarily discussed in the context of an emergency response to the COVID-19 pandemic rather than as a standard practice approach implemented under non-pandemic conditions.

### A sense of safety and control — ‘it puts the care back into the control of the consumer’

Healthcare professionals in this study observed that being able to access telehealth from the comfort of their own home gave some clients a greater sense of safety, as “they’re able to be in their own space where they probably feel more safe” and they “don’t need to look someone in the eye or feel awkward.” One participant noted that this sense of safety facilitated greater openness by her clients, enabling them to “say what they really felt”. However, participants also expressed concern about the use of eHealth with clients whose home environment is unsafe, as online or telephone consultations do not always afford the “privacy to be able to have the conversations that [clients] wanted to be having.”

Several participants observed that telehealth increased clients’ control over the healthcare interaction, allowing them to decide “whether they hang up or not, rather than needing to walk out of a room or leave a space”. In particular, digital health interventions in the form of apps gave clients more control over the recovery process, enabling them to access care when and how they most needed it:

“eHealth puts the care back into the control of the consumer. So rather than waiting for the next psychology appointment or anything like that, it allows the recovery process to be back, a little bit more in control of when the consumer needs it and they can then direct either how quickly they might do a module through an app, or whether there’s a mindfulness practice that they grab off a different app and they can do that when they actually need it in live time.” (SM01, social work manager).

In contrast, health professionals experienced a loss of control when using eHealth technologies which, for some, resulted in a feeling of discomfort:

“You then realise like wow we really want control. We want to make sure that we can be there, and we can support you, and we think that that can only happen that [one] way” (CL03, social worker).

Several participants described the sense of unease created by the disruption to standard clinical procedures and therapeutic approaches during the rapid and “forced” implementation of eHealth during COVID-19, and the subsequent lack of control clinicians experienced over the assessment process. One participant noted that her clinical colleagues “didn’t feel like their assessments were a true reflection of what was happening”. In fact, some healthcare professionals who were working with “the most unwell people in our community” “refused to do initial assessments [using telehealth] because of the concerns about missing something”. One participant observed that the level of resistance to eHealth is dependent on the perceived risks it poses to quality of the healthcare assessment:

“I think there’s probably more resistance for different people, different teams depending on their comfortability [sic] with technology or the risk that they might perceive might happen by missing something if you’re not in the room” (CL09, social worker).

### Expanding the breadth of care — ‘the stuff in between’

Healthcare professionals in this study observed that the “flexibility” and “convenience” of eHealth technologies enabled them to “reach out to a much larger audience” and facilitate participation by people who may not otherwise have access to healthcare services:

“So, they don’t have to be in the clinical space in a particular space to participate in the traditional health service or any discussion, they can be anywhere and that gives a chance for a larger group of people to participate. And the feeling to participate I think as well.” (CL08, nurse).

Participants observed that eHealth is most likely to facilitate broad participation and inclusion when it is “adapted to different groups of consumers … whether that’s differences in gender or age or culture or, yeah, or what that person’s actually experiencing”. Where an eHealth tool was recommended by health professionals, but did not meet the needs of clients, participants noted that “[clients] deleted it from their phones because they never use it”. For example, one participant noted that an app had been rolled out across their clinic but had been deleted by “over half of patients” because they “don’t want it”. The same participant acknowledged that, while client interest in eHealth apps is important, also crucial is that clinicians are “invested in it” and “think it works”.

Several participants acknowledged that “clinicians are only available between certain times during the day” and that “concerns about mental health and low moods don’t stop at 4:30”, noting that “some sort of [eHealth] support could be really important at these times”. These participants tended to view eHealth as “the stuff in between” more traditional forms of healthcare provision, rather than as a standalone form of care.

### Resources and capacity — ‘it’s kind of dependent on someone’s resources’

A key finding from this study was that clients’ engagement with eHealth tools is often impacted by a lack of resources. Participants noted that “many people don’t have computers and laptops and things like that in their home” or may have difficulty maintaining “consistent” internet or phone access as “they may not pay their phone bill all the time”. Even when clients are more financially stable, spending money on eHealth technologies “may not necessarily be a priority” for them. Consequently, one participant noted that “if you want to make [eHealth] broadly available to patients, it’s got to be free.”

A lack of capacity and technological literacy were also highlighted as key barriers to the uptake of eHealth by clients. As one health professional noted:

“A lot of our clients, they lose their phones, or they break their phones, or they just don’t have the capacity, or sometimes they just don’t have that understanding how technology works.” (CL02, social worker).

Several participants observed that “the most marginalised people in our community” (i.e., those from “lower socioeconomic backgrounds and with the lowest IT literacy”) are also the most likely to experience difficulties in accessing eHealth tools. Participants perceived multiple, often intersecting, barriers that impacted on their clients’ access to eHealth, including cognitive ability, mental illness, and literacy. As one participant summarised:

“The more unwell someone is, the more challenges they have in accessing eHealth options. I think just the processes involved in maintaining a mobile phone account can be really difficult.” (CL04, nurse).

Participants also emphasised the lack of support available to help clients engage with eHealth tools:

“We’ve been provided with support and training in how to document, set up Skype, use Skype and troubleshoot stuff. Whereas when we call clients, it’s like, all right, we’re going to send you a link. If it doesn’t work, that’s it – there’s no troubleshooting support for them.” (CL02, social worker).

Some health professionals tried to address this issue by providing technical support to clients themselves, which led to a significant increase in their own workload:

“The biggest workload for our team involved setting up clients in being able to access telehealth. Having a social worker and/or nurse joining a doctor for those three clinics was about just getting clients to the appointments. So, we did a lot of phone calls leading up the appointment.” (CL12, social worker).

### A fundamental shift— ‘it does require the practitioner to try a whole new mode’

Overall, healthcare professionals recognised the need for healthcare services to embrace technology as “the way of the future”:

“I mean we’re living in a technology world aren’t we so we’re going to have to progress.” (CL07, nurse).

However, a number of participants described feelings of “hesitancy”, “scepticism” and “discomfort” in relation to eHealth, questioning whether “it could be equivalent or better than meeting face to face.” Participants who expressed these sentiments tended to describe eHealth as an insufficient ‘replacement’ of face-to-face practice:

“I mean we’ve had telehealth for many years, but health professionals are sceptical that it could be equivalent or better than meeting face to face.” (SM02, social work manager).

For example, in describing her hesitancy to integrate eHealth into her practice, one participant expressed a concern about losing a valued aspect of her practice:

“I really value the face-to-face aspect of getting to know someone. I’ve been a bit hesitant, I guess, to introduce eHealth to my practice.” (CL02, social worker).

Another participant described eHealth as disrupting the “etiquette” and “seriousness” of healthcare provision:

“I think the families are not taking it serious enough, so that lack of etiquette, like walking into the teenager’s bedroom carrying the phone, and waking the kids up in bed, or the kids aren’t even there, the kids are playing outside. Yeah. Not having the gear uploaded, like not taking it as seriously as if they came to the clinic.” (CL16, nurse).

When discussing her colleagues’ scepticism about eHealth, one participant noted that the use of eHealth requires a fundamental shift in the way healthcare professionals engage and interact with clients:

“They’re sceptical about the technology. I think it you know, it does, you know, require the practitioner to try a whole new mode of speaking with people and learning that technology, even though it’s not that complicated.” (SM02, social work manager).

This participant concluded that “I guess they’re sceptical [because] I think they feel really uncomfortable not being able to have full verbal and non-verbal communication.” However, some participants also described positive experiences using eHealth, and emphasised the benefits of this mode of delivery for their clients:

“Some clinicians did feel like the [telehealth] service offered during COVID was more superficial in terms of the intervention you could do because the person might be distracted. I haven’t found that myself. The clients I have worked with, as I said, it kind of has worked in their favour and even if there has been challenges in the environment they do try to manage those so they can be present.” (CL15, social worker).

These participants often recounted aspects of eHealth that were “different”, but that extended their practice or even made it more “powerful”:

“There can be opportunities to learn about your patient too. When you see them in their home it’s different to seeing them in the room … you get a bit of an insight into their life or what they’re interested in. So, you also learn about them more.” (CL01, psychiatrist).

“Sometimes the phone can be more, I can feel more connected because I’m not distracted, I mean I’m looking for an emotional assessment, the assessment of their emotional state as well as a connection, sometimes the phone can be even a bit more powerful in that way.” (CL09, social worker).

## Discussion

We reported on a subset of qualitative findings from interviews with multidisciplinary health professionals with the aim of aim of examining issues of equity and justice in the implementation and uptake of eHealth technologies in practice. In the discussion, we apply Fraser’s [24] multidimensional conception of social justice to identify key dimensions and patterns of *distribution*, *recognition*, and *participation* in the implementation and uptake of digital health services. By deliberately situating our findings within this social justice frame, our study responds to Brownson and colleagues’ [2] call for the need to make health equity the highest priority in implementation science.

Health professionals in this study highlighted the empowering effect eHealth had on their clients, offering them a greater sense of safety, allowing them to determine the nature and pace of their healthcare, and ultimately, giving them more control over their own treatment and recovery. Applying Fraser’s social justice framework, this finding suggests that eHealth has the potential to enhance clients’ *recognition*, enabling them to participate as full or equal partners in the clinical interaction. However, health professionals also expressed concerns about the use of eHealth with clients whose home environment is unsafe, noting that online and telephone consultations did not offer a secure and private space for these clients to discuss their experiences and needs. This finding suggests that the use of some forms of eHealth may inadvertently reinforce existing ‘injustices of recognition’ [24], such as those caused by domestic violence, by removing clients’ access to a safe clinical space in which their voices can be heard.

The use of eHealth, and particularly telehealth, was perceived by many healthcare professionals as reducing their control over the healthcare interaction, which often resulted in feelings of discomfort or unease. Some participants recognised and accepted these feelings as a necessary consequence of letting go of control. Others responded with resistance or simply refused to use eHealth technologies, citing their lack of control over the assessment process as an unacceptable “risk” to client safety. It is important to note that clients were generally not consulted about the decision to use, or not use, eHealth, reflecting a lack of *representation* of their voices in decision making relating to their healthcare. Of interest, was the observation by one healthcare professional that eHealth technologies disturb the “etiquette” and “seriousness” of healthcare provision, suggesting that professional resistance to eHealth implementation may be driven not only by concerns about client risk and safety, but by a fundamental unease about the threat eHealth poses to the rituals, norms, and power structures inherent within traditional healthcare systems and processes. This finding is consistent with previous research, which highlights the professional cultures, power and sociohistorical forms of legitimacy that underpin healthcare systems, and that privilege certain views and approaches over others [37]. It highlights the critical need for implementation science to move beyond a basic focus on individual attitudes and beliefs about eHealth to illuminate the underlying forces; the social, historical, and cultural factors that determine which voices are privileged, and which are misrepresented, in the decision to use or refuse eHealth.

Many healthcare professionals in this study perceived eHealth as “flexible” and “convenient” and therefore able to reach a “much larger audience”. Applying Fraser’s social justice framework, this finding suggests that eHealth has the potential to enhance the *distribution* of healthcare. To facilitate broad participation, healthcare professionals noted that eHealth tools need to be adaptable to the needs and circumstances of diverse groups, including people for whom telehealth may not provide a “safe” space. Participants also reported that clients who were most marginalised and disadvantaged also experienced the greatest difficulties in accessing eHealth, highlighting a lack of organisational support to help clients engage with eHealth tools. Some clinical teams tried to address this service gap themselves by providing additional assistance to clients, often at a significant perceived cost to their own workload. These findings underscore previous research highlighting critical inequalities in the distribution of eHealth services [17]. However, application of Fraser’s framework facilitates a move beyond conventional arguments that digital health *itself* perpetuates healthcare disparities [16], to identify the underlying structures that hinder the equitable distribution of eHealth resources.

Fraser [23] differentiates between two main strategies for pursuing distributive justice: (a) *affirmative* and (b) *transformative*. The former focuses on ‘correcting inequitable outcomes without disturbing the underlying framework that generates them’, while the latter seeks to critically challenge the ‘deep structures’ of value that determine the distribution of social goods and services [23]. From this perspective, implementation efforts cannot afford to ignore the deeper health systems and bureaucratic structures that shape the distribution of eHealth resources. Further, we must move beyond a critique of these ‘existing power structures’ to begin ‘imagining alternatives’ [24]; exploring implementation practices that contribute to the formation, development and nurturing of systems that maximise the potential of eHealth to enhance health equity.

### Limitations

The central limitation of this study is that only multidisciplinary health professionals were interviewed, so one side of the story is missing. Thus, ironically, our study itself reflects a lack of *representation* of service user voices. However, our findings also suggest that a focus on equity in eHealth implementation must identify the actions, beliefs and attitudes of health professionals that may unintentionally perpetuate inequity in eHealth engagement, as well as the price health professionals can pay when they try to facilitate implementation efforts that are not well supported at a wider organisational level. This article, thus, offers an important starting point for future implementation research that considers questions of equity and justice from a service user perspective. A further limitation is that some study participants were known by the interviewer, which could have affected the results [38]. However, it is also likely that the pre-existing trust and rapport established with participants may have enhanced the information gathering process. Additionally, the included participants represented a broad group of clinicians and service managers working with a variety of different client groups in health and mental health contexts. It is therefore important to acknowledge that there are distinct occupational differences for each participant and the findings should not be generalised. Lastly, in this study we adopted a broad definition of eHealth, which meant that it was not always possible to identify which form of eHealth participants were referring to. Future research should employ a more nuanced approach to examine the relationship between specific forms of eHealth and health equity.

## Conclusion

We investigated the perceptions of multidisciplinary health professionals on the implementation and uptake of eHealth in clinical practice and reported on a subset of findings with the aim of examining issues of equity and justice in the implementation and uptake of eHealth technologies in practice. Application of a social justice lens revealed critical systemic and cultural barriers that hinder the representation of clients’ voices in the decision to use eHealth technologies and perpetuate inequalities in the distribution of eHealth services. Findings highlight the need for implementation science to focus more upstream, complementing individually oriented approaches with a focus on wider social, policy or organisational change.

## Supplementary Information

**Additional file 1.** Consolidated criteria for reporting qualitative studies (COREQ): 32-item checklist.

**Additional file 2.** Interview Schedule for eCliPSE Pilot Study – Qualitative Interviews with Clinicians and Practice Managers.

## Data Availability

The datasets used and/or analysed during the current study are available from the corresponding author on reasonable request.
