# Supplementary material for: Placing equity at the heart of eHealth implementation: a qualitative pilot study
Source: Int J Equity Health. 2022 Mar 18;21:38. doi: 10.1186/s12939-022-01640-5 (PMC8931179; doi:10.1186/s12939-022-01640-5)
Supplement: Supplementary file 1 — Additional file 1. Consolidated criteria for reporting qualitative studies (COREQ): 32-item checklist. [file 12939_2022_1640_MOESM1_ESM.docx]

**Additional File 1**

**Consolidated criteria for reporting qualitative studies (COREQ): 32-item checklist**

| **Domain 1: Research team and reflexivity** | | | |
| --- | --- | --- | --- |
| **Personal characteristics** | | | |
| 1. | Interviewer/facilitator | Which author/s conducted the interview or focus group? | p. 6 |
| 2. | Credentials | What were the researcher’s credentials? | p. 6 |
| 3. | Occupation | What was their occupation at the time of the study? | p. 6 |
| 4. | Gender | Was the researcher male or female? | p. 6 |
| 5. | Experience and training | What experience or training did the researcher have? | p. 6 |
| **Relationship with participants** | | | |
| 6. | Relationship established | Was a relationship established prior to study commencement? | p. 6 |
| 7. | Participant knowledge of the interviewer | What did the participants know about the researcher? | p. 6 |
| 8. | Interviewer characteristics | What characteristics were reported about the interviewer/facilitator? | n/a |
| **Domain 2: Study design** | | | |
| **Theoretical framework** | | | |
| 9. | Methodological orientation and theory | What methodological orientation was stated to underpin the study? | p. 7 |
| **Participant selection** | | | |
| 10. | Sampling | How were participants selected? | p. 5 |
| 11. | Method of approach | How were participants approached? | p. 5 |
| 12. | Sample size | How many participants were in the study? | p. 5 |
| 13. | Non-participation | How many people refused to participate or dropped out? | n/a |
| **Setting** | | | |
| 14. | Setting of data collection | Where was the data collected? | p. 6 |
| 15. | Presence of non-participants | Was anyone else present besides the participants and researchers? | n/a |
| 16. | Description of sample | What are the important characteristics of the sample? | p. 6 |
| **Data collection** | | | |
| 17. | Interview guide | Were questions, prompts, guides provided by the authors? Was it pilot tested? | p. 6-7 |
| 18. | Repeat interviews | Were repeat interviews carried out? If yes, how many? | n/a |
| 19. | Audio/visual recording | Did the researcher use audio or visual recording to collect the date? | p. 7 |
| 20. | Field notes | Were field notes made during and/or after the interview or focus group? | n/a |
| 21. | Duration | What was the duration of the interviews or focus group? | p. 6 |
| 22. | Data saturation | Was data saturation discussed? | p. 5 |
| 23. | Transcriptions returned | Were transcripts returned to participants for comment and/or correction? | n/a |
| **Domain 3: Analysis and findings** | | | |
| **Data analysis** | | | |
| 24. | Number of data coders | How many data coders coded the data? | p. 7 |
| 25. | Description of coding tree | Did authors provide a description of the coding tree? | n/a |
| 26. | Derivation of theme | Were themes identified in advance or derived from the data? | p. 7 |
| 27. | Software | What software, if applicable, was used to manage the data? | p. 7 |
| 28. | Participant checking | Did participants provide feedback on the findings? | n/a |
| **Reporting** | | | |
| 29. | Quotations presented | Were participant quotations presented to illustrate the themes / findings? Was each quotation identified? | p. 8-14 |
| 30. | Data and findings consistent | Was there consistency between the data presented and the findings? | p. 8-14 |
| 31. | Clarity of major themes | Were major themes clearly presented in the findings? | p. 8-14 |
| 32 | Clarity of minor themes | Is there a description of diverse cases or discussion of minor themes? | p. 8-14 |
