# Supplementary material for: Placing equity at the heart of eHealth implementation: a qualitative pilot study
Source: Int J Equity Health. 2022 Mar 18;21:38. doi: 10.1186/s12939-022-01640-5 (PMC8931179; doi:10.1186/s12939-022-01640-5)
Supplement: Supplementary file 2 — Additional file 2. Interview Schedule for eCliPSE Pilot Study – Qualitative Interviews with Clinicians and Practice Managers. [file 12939_2022_1640_MOESM2_ESM.docx]

**Additional File 2**

***Interview Schedule for eCliPSE Pilot Study – Qualitative Interviews with Clinicians and Practice Managers***

**Introduction:**

At the start of the focus groups and interviews, the researcher will confirm with participants that they have read and understood the participant information statement. A brief overview of the study will be provided, including participants’ rights to withdraw from the focus groups and interviews at any time. The researcher will then ask participants to confirm that they still wish to participate in the study by gaining verbal consent, which will be audio-recorded. The questions below may be modified slightly, asked with discretion or in a slightly different order, dependent on the direction of each focus group/interview.

**Questions:**

1. Can you tell me about your current role and practice?
2. eHealth is becoming a major government priority. What are your thoughts on the use of electronic support tools as part of patient care in the mental health, alcohol and other drug services, and healthcare arena?
3. Do you currently use any form of eHealth applications in your work (or as part of your patient care plans)? If yes: how often? Can you share those experiences? If no: have you been interested to use it, but it wasn’t used in your organisation, and why?
4. What factors do you think should be in place within your clinical setting to support eHealth implementation? Prompts:
   1. Do you feel that the clinical setting within your workplace is supportive of eHealth implementation?
   2. Do you feel that enough support, training, and resources are provided by your organisation for eHealth applications?
   3. What factors (resources, information, support etc.) should be in place to support the effective implementation of eHealth interventions?
   4. What strategies can organisations employ to overcome barriers to implementation?
   5. What are the drivers that might facilitate the implementation of eHealth applications in your organisation and/or as part of the care you provide to your patients?
5. Is there anything else you would like us to know about the implementation of eHealth applications in your service? E.g., what has been/would be most helpful? What have been/ would be the greatest barriers?
